# Associations Between Instrumented Mouthguard-Measured Head Acceleration Events and Post-Match Biomarkers of Astroglial and Axonal Injury in Male Amateur Australian Football Players

**DOI:** 10.1007/s40279-024-02138-6

**Published:** 2024-11-19

**Authors:** Lauren J. Evans, William T. O’Brien, Gershon Spitz, Steven Mutimer, Becca Xie, Lauren P. Giesler, Brendan P. Major, James W. Hickey, Spencer S. H. Roberts, Biswadev Mitra, Terence J. O’Brien, Sandy R. Shultz, Stuart J. McDonald

**Affiliations:** 1 Department of Neuroscience, Central Clinical School, Monash University, 99 Commercial Road, Melbourne, VIC Australia; 2 Monash-Epworth Rehabilitation Research Centre, School of Psychological Sciences, Monash University, Clayton, VIC Australia; 3 School of Exercise and Nutrition Sciences, Deakin University, Burwood, VIC Australia; 4 Emergency and Trauma Centre, The Alfred Hospital, Melbourne, VIC Australia; 5 School of Public Health and Preventive Medicine, Monash University, Melbourne, VIC Australia; 6 Department of Neurology, The Alfred Hospital, Melbourne, VIC Australia; 7 Department of Medicine, Royal Melbourne Hospital, The University of Melbourne, Parkville, VIC Australia; 8 Centre for Trauma & Mental Health Research, Vancouver Island University, Nanaimo, BC Canada

## Abstract

**Background:**

Advances in instrumented mouthguards (iMGs) allow for accurate quantification of single high-acceleration head impacts and cumulative head acceleration exposure in collision sports. However, relationships between these measures and risk of brain cell injury remain unclear.

**Aim:**

The purpose of this study was to quantify measures of non-concussive head impact exposure and assess their association with blood glial fibrillary acidic protein (GFAP), neurofilament light (NfL) and phosphorylated-tau-181 (p-tau-181) levels in male Australian football players.

**Methods:**

A total of 31 athletes underwent in-season (24 h post-match) and post-season (> 5 weeks) blood collections and/or wore HITIQ Nexus A9 iMGs measuring peak linear (PLA) and rotational (PRA) acceleration. Match footage was used to verify and code impacts. Blood GFAP, NfL, and p-tau-181 were quantified using Simoa and natural log transformed for analysis. Associations between post-match biomarkers and within match maximum single impact and cumulative PLA/PRA were assessed with linear mixed models.

**Results:**

In-season versus post-season elevations were found for GFAP (mean difference 0.14, 95% CI 0.01–0.26, *p* = 0.033), NfL (mean difference = 0.21, 95% CI 0.09–0.32, *p* = 0.001) and p-tau-181 (mean difference = 0.49, 95% CI 0.33–0.65, *p* < 0.001). Post-match GFAP was associated with maximum single impact PLA (*B* = 0.003, 95% CI 0.0002–0.005, *p* = 0.036), cumulative PLA (*B* = 0.001, 95% CI 0.0002–0.002, *p* = 0.017), cumulative PRA (*B* = 0.01, 95% CI 0.002–0.02, *p* = 0.014), and impact number (*B* = 0.03, 95% CI 0.003–0.05, *p* = 0.029) within a single match. Change in NfL levels between two-matches correlated with cumulative PLA (*r* = 0.80, 95% CI 0.38–0.95, *p* = 0.005), PRA (*r* = 0.71, 95% CI 0.19–0.92, *p* = 0.019) and impact number (*r* = 0.63, 95% CI 0.05–0.89, *p* = 0.038).

**Conclusion:**

Maximum and cumulative head accelerations in Australian football, measured by iMGs, were associated with elevated blood biomarkers of brain injury, highlighting the potential of both technologies for head impact management in collision sports.

**Supplementary Information:**

The online version contains supplementary material available at 10.1007/s40279-024-02138-6.

## Key Points

**Table:** 

Instrumented mouthguard measures of maximum and cumulative non-concussive head accelerations within a single Australian football match were linked to post-match elevations in the brain-specific blood biomarker glial fibrillary acidic protein (GFAP).	
Cumulative head acceleration totals over a two-match period were associated with increases in blood levels of the axonal injury marker neurofilament light (NfL).	
These findings emphasise the potential benefits of integrating instrumented mouthguards and blood biomarkers for monitoring head impact exposure and assessing potential neurobiological effects in collision sports.	

## Introduction

The global sporting and research community has increasingly acknowledged the potential long-term neuropathological consequences of repeated head acceleration events (HAEs) in collision sports. ‘Non-concussive’ or ‘sub-concussive’ impacts involve contact to the head, neck or body, resulting in rapid acceleration and deceleration of the brain without clinical signs and symptoms [1–3]. Extensive exposure to repeated HAEs in collision sports is linked to increased risk of neurodegenerative diseases such as chronic traumatic encephalopathy (CTE) and Alzheimer’s disease [4, 5]. Existing research on the potential short- and long-term effects of HAEs in collision sports has often relied on surrogate measures of exposure, such as career duration, level of participation, and playing position. Indeed, these measures have merit, and recent retrospective studies utilising them have significantly increased our understanding of the potential cumulative effects of HAEs [4–10], although most lack measures related to the neurobiological mechanisms underlying these effects.

Recent strides in accelerometry technology, notably the use of instrumented mouthguards (iMGs) tailored for optimal skull coupling through fitting to the upper dentition [11, 12], offer a more precise and reliable quantification of HAE exposure for prospective studies. Combined with immunoassays capable of detecting subtle increases in blood biomarkers indicative of brain cell trauma [13, 14], this provides a powerful approach to investigate HAEs and the associated neurobiological changes in individual athletes.

Studies utilising accelerometry have suggested that the cumulative magnitude of HAE exposure in collision sports, especially over extended periods, may be linked to increases in blood-based biomarkers of brain cell trauma [15–20]. However, several limitations persist, leaving crucial knowledge gaps. First, accelerometry technology has advanced with higher sampling rates, lower thresholds to trigger recordings, and specific classification algorithms to filter out noise [11, 12, 21]. Consequently, previous studies, particularly those lacking video verification, may not accurately quantify associations with biomarkers. Second, biomarkers have diverse temporal profiles after sport-related concussion (SRC) [14, 22–27], and there is evidence that levels of some markers can be influenced by exercise alone, with changes highly dependent on the time elapsed since exercise [24, 28–30]. Hence, careful selection and implementation of the blood sampling times is likely crucial. Thirdly, studies to date linking HAEs and biomarkers have largely focussed on American football. It is highly likely that the nature of exposure is different in sports such as Australian football, where disparities in HAE number, frequency, magnitude and direction, together with the absence of helmets, may contribute to variations in kinematics and associated biomarker changes [31]. Finally, our understanding of the effects of short-term exposure, such as in a single match, remains limited. Similarly, the uncertainty surrounding whether a single high-magnitude non-concussive impact is as likely to induce neurobiological changes as multiple smaller impacts is significant and warrants investigation. These two factors are key to informing how objective tools can be effectively employed to enhance head impact management in sports.

In our investigation of amateur Australian football players, we utilised iMGs with video verification to quantify short-term HAE exposure, and blood sampling at 24 h after matches and > 5 weeks post-season to quantify biomarkers of astroglial and axonal injury. We strategically chose to collect blood at 24 h post-match, aligning with the known peak of GFAP levels after mild traumatic brain injury and SRC [32–34], a top-performing brain-specific diagnostic candidate in these contexts [14]. Additionally, blood NfL levels do not immediately peak after brain injury, but instead several days or weeks later [14, 22, 33], reflecting a prolonged brain clearance and circulating half-life. We posited that this kinetic profile following repeated non-concussive impacts would enable the summation of NfL levels over the season, and consequently that NfL levels at 24 h post-match might not strongly correlate with HAEs in the directly preceding match. Instead, a more appropriate measure for assessing NfL associations with recent HAE exposure would involve tracking changes over time. Finally, we explored the measurement of phosphorylated tau 181 (p-tau-181), a blood biomarker linked with tau pathology [35].

Our aims were to: (1) characterise HAE exposure in amateur adult men’s Australian football participants; (2) compare in-season and post-season blood levels of GFAP, NfL, and p-tau-181; (3) investigate the associations between post-match biomarkers and HAE measurements of: (a) the number of head impacts, (b) the cumulative PLA/PRA (i.e. sum of all HAEs in a match) and (c) the maximum PLA/PRA (i.e. largest single impact acceleration values within a match); and (4) determine whether cumulative impact PLA/PRA and HAE number over two consecutive matches correlate with change in NfL levels over this period. Aim 3 was the primary aim of this study.

We hypothesised that: (1) tackles would account for the most HAEs; (2) blood levels of GFAP, NfL, and p-tau-181 would be elevated in-season; (3) post-match GFAP levels would be most strongly associated with the cumulative and maximum PLA/PRA; and (4) change in NfL levels over two consecutive matches would be associated with cumulative PLA/PRA and HAE number.

## Methods

### Study Design

**Fig. 1:**
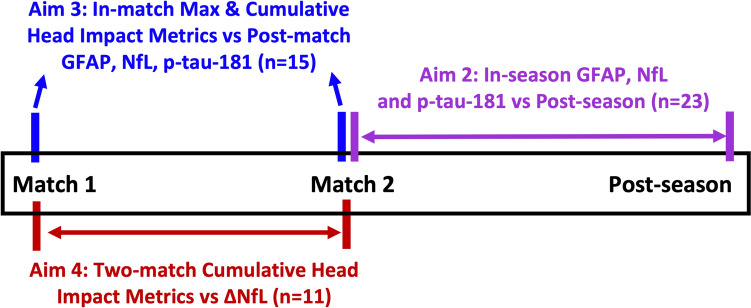
Study design summary for Aim 2–4. Aim 2 involved collecting paired in-season and post-season blood samples from 23 players. For Aim 2, the in-season sample was taken after the first instance a player had a post-match blood sample following two consecutive matches, including 11 players with iMG data and 12 players without iMG data. Aim 3 focused on 15 players who wore iMGs and provided blood samples 24 hours post-match for up to two matches; data from these matches were analyzed, resulting in 27 paired iMG and blood data points, with three players contributing data from only one match. Aim 4 included 11 players who wore iMGs and provided post-match blood samples over two consecutive weeks. Note that the in-season sample for Aim 2 was not necessarily from Match 2 as in Aims 3 or 4, but rather the first post-match sample obtained after playing two consecutive matches

A total of 41 male Australian footballers from nine Victorian Amateur Football Association (VAFA) clubs were recruited to participate as part of a larger SRC-focussed program featuring serial blood and symptom measurements [27]. From these, 26 players from two clubs underwent three-dimensional (3D) dental scans to create custom-fitted HITIQ Nexus A9 Instrumented Mouthguards (HITIQ Pty. Ltd., Melbourne, VIC, Australia). Using pre-defined criteria, data from 32 of the players contributed to at least one of the four aims in the current study (Fig. 1). Team physiotherapists and/or sports trainers were present at all matches and screened for SRC using the Sport Concussion Assessment Tool 5; matches involving iMG use were overseen by experienced physiotherapists.

For aim 1 (i.e. HAE characterisation), players were required to have participated in at least one full VAFA match that was filmed to enable video verification while wearing the iMG.

For aim 2 (i.e. in-season versus post-season biomarkers), players must have had a blood sample taken 24 h after their second consecutive played match (i.e. two consecutive Saturdays; a single sample of the first such occurrence used for analysis), and a single post-season sample collected at least 4 weeks after the completion of the season. This method was used to capture biomarker levels after a relatively consistent period of match exposure before the in-season sample and a relatively consistent period of no matches before the post-season sample.

For aim 3 (i.e. single match HAE versus biomarkers), players were required to have participated in at least one filmed match while wearing the iMG and had 24 h post-match blood collected, with a maximum of two-paired matches per player used, and the first two such instances in the season for each player used for analysis.

In aim 4 (i.e. two match HAE exposure vs change in NfL), data were composed of individuals who played in two consecutive filmed matches while wearing the iMG, with blood collected 24 h after both matches. The rationale for this approach was that the delayed release of NfL into circulation, combined with the potential summation of serum NfL levels due to impacts over several weeks owing to its long half-life, would mean that the sample taken post-match 1 would serve as a more suitable baseline reference point for assessing NfL elevation than absolute NfL concentration. Although our expectation was that the change in NfL over two consecutive matches (i.e. ΔNfL) would therefore primarily be driven by HAE exposure in match 1, we also included HAEs in match 2 to account for (likely smaller) NfL elevations at 24 h.

Exclusion criteria at enrolment was a concussion or major musculoskeletal injury (e.g. fracture, dislocation) in the preceding 6 months, history of moderate or severe TBI, a medical history that may contribute to neurological impairment (e.g. epilepsy, dementia, multiple sclerosis), current dental or orthodontic appliances of the upper dentition (e.g. braces), or current use of antiplatelet or anticoagulant agents. During the study, individuals who were diagnosed with a SRC ceased involvement in blood collections for aims 2–4 from that point forward (i.e. aims 2–4 contain data only from head impacts not resulting in SRC).

### Baseline Surveys

After providing written consent, participants completed a series of questionnaires related to their medical and sporting history. This was conducted in-person at the time of dental scanning for iMG production, or for non-iMG participants, at the time of the first post-match blood collection.

### iMGs and HAE Quantification

The HITIQ Nexus A9 iMG employed three tri-axial accelerometers and one tri-axial gyroscope. Tri-axial accelerometers were sampled at roughly 3200 Hz, while the gyroscope sampling rate was set at roughly 800 Hz. Due to individual variation between accelerometer sampling rates, time series for each axis of the three linear accelerometer sensors were resampled to exactly 3200 Hz. Gyroscope data were up-sampled from 800 to 3200 Hz. A fourth-order zero-phase Butterworth filter was utilised to filter all signals, with a cut-off frequency set at 300 Hz [36]. Most metrics for head injuries are based on the measurements of linear acceleration, rotational velocity, and rotational acceleration taken at the estimated centre of gravity (CoG) of the head. CoG values were computed under the rigid body assumption and the associated kinematic relationships from the measurements made at iMG. Under the rigid body assumption, the rotational velocity and acceleration recorded at any point will match those at the CoG. Moreover, the following equation governs the relationship between the linear acceleration at the CoG and any arbitrary point A on the head:

$$aCoG=aA+a\times rA+\omega \times \left(\omega \times rA\right),$$

where aCoG is the linear acceleration at CoG, aA is the linear acceleration recorded at point A, $\omega$ is the rotational velocity, $a$ is the rotational acceleration and rA is the vector from point A to CoG. With three linear accelerometers, the equation expands to:

$$aCo{G}_{1}=a{a}_{ ac{c}_{1}}+a\times {r}_{ ac{c}_{1}}+\omega \times (\omega \times {r}_{ ac{c}_{1}})$$

$$a{CoG}_{2}={aa}_{ acc2}+a\times {r}_{ ac{c}_{2}}+\omega \times (\omega \times {r}_{ ac{c}_{2}})$$

$$aCo{G}_{3}={aa}_{ acc3}+a\times {r}_{ acc3}+\omega \times (\omega \times {r}_{ ac{c}_{3}})$$

where aCoG_i_ is the linear acceleration at CoG calculated from accelerometer i, aacc_i_ is the linear acceleration at and measured by accelerometer i and racc_i_ is the vector from accelerometer i to the CoG. Linear acceleration at CoG aCoG is then calculated using the mean of the three CoG estimates:

$$aCoG = (aCo{G}_{1}+aCo{G}_{2} + aCo{G}_{3})/3.$$

It should be noted that all parameters in the equations conform to the SAEJ211 standard coordinate system. The rotational matrix, which is necessary for transforming the data from each sensor’s local coordinate to the SAEJ211 standard coordinate system, along with the racc_i_ vector, was precisely calibrated for each mouthguard using a proprietary calibration procedure. In the calibration procedure the CoG was defined as the 50th percentile male head [37]. Peak linear and rotational metrics were calculated taking the resultant of each kinematic measurement.

Before each match, club medical and training staff synchronised the iMGs to the current date and time and created a recording session on the HITIQ Nexus application, with each session set to automatically begin recording 15 min prior to match starting and 30 min after the expected finish. After the completion of each match, club staff uploaded iMG data to a Nexus portal for instant data processing.

Publicly available video footage from 32 home and away matches (single camera angle following match play, 720p, 60fps) was used to locate impacts using capture timestamps recorded by the iMG. Any recorded HAEs not captured on game footage, or which occurred during a break in play (i.e. quarter breaks, pre- or post-game) were excluded from analysis. Impacts were located on the footage and coded as true-positive (i.e. video shows player experiences visible HAE from a contact event at the iMG-triggered timestamp) or false positive (i.e. video shows player experiences no visible HAE from a contact event at the iMG-triggered timestamp). Timestamps of each true- and false-positive impact were located and verified by the same rater (LJE). True-positive impacts were further coded to detail impact location and match play situation (e.g. head to ground, tackle). Any impact in which the player was not on camera at the time of iMG capture was coded as ‘off camera’ and not included in further analyses.

### Blood Collection and Serum Analyses

Venous blood was collected into BD Vacutainer^®^ SST™ II Advance tubes for serum preparation, and BD Vacutainer^®^ EDTA tubes for plasma preparation. Serum tubes were inverted several times and allowed to clot at room temperature for 30 min prior to centrifugation at 1500 *g* for 10 min. Plasma tubes were inverted several times and stored at 4 °C prior to centrifugation at 1100 *g* for 10 min. Samples were transferred to aliquots, flash-frozen, and stored at − 80 °C. Quantification of serum GFAP and NfL was conducted using Simoa Neurology 2-Plex B kits, and plasma p-tau-181 using Advantage V2 kits on the SIMOA HD-X Analyzer (Quanterix Corp., Billerica MA, USA). All samples were tested in duplicate with an average coefficient of variation of 7.38%, 7.34% and 5.10% for GFAP, NfL and p-tau-181, respectively. All samples measured above the lower limit of detection (GFAP: 0.475 pg/mL, NfL: 0.065 pg/mL and p-tau-181: 0.028 pg/mL).

### Statistical Analyses

Descriptive statistics were used for aim 1 on head impact frequency, magnitude and match play coding. A positive predictive value (PPV) was calculated for true-positive and false-positive captures (PPV = true-positives/true-positives + false-positives). Density plots were used to assess the normality of biomarker concentration. A natural logarithm (Ln) transformation was consequently applied to all biomarker concentrations for aims 2–3. For aim 2, paired Student’s *t*-tests were used to compare biomarker levels in-season versus post-season. For aim 3, linear mixed models with fixed effects of match number, age and body mass index were used to determine the association between post-match blood biomarker levels and five measures: (1) cumulative PLA, (2) cumulative PRA, (3) match total impact number, (4) maximum single impact PLA and (5) maximum single impact PRA. Participant was entered as a random term in all linear mixed effects models. For aim 4, we assessed the association between ΔNfL values (representing the raw concentration difference between NfL post-match 2 and NfL post-match 1) and cumulative PLA, PRA and impact number over the two matches using Spearman’s *r*. Assumptions for all models were assessed with the performance package in R. Sample sizes for all aims were derived from a convenience sample. Significance level was set at *p* < 0.05. Statistical analyses were conducted in R (Version 4.2.2) and GraphPad Prism (Version 9.3.1).

## Results

### Demographics

Overall study demographics are outlined in Table 1, with highly comparable demographics found for each aim. Aim 1 featured 23 players who participated in at least one filmed match while wearing an iMG, with a total of 156 player matches available for analysis. Aim 2 featured 23 players that completed in-season and post-season blood collections. Aim 3 had 15 players who participated in a filmed match while wearing an iMG in either 2 (*n* = 12) or 1 (*n* = 3) match with corresponding post-match blood collections, resulting in a total of 27 iMG and blood data pairs. Aim 4 featured 11 players who participated in two consecutive filmed matches with an iMG and blood collections performed after each match, resulting in 22 data pairs. Details of the 31 participants and match participation, iMG use and blood collection are outlined in Online Resource 1.

**Table 1:** Participant demographics

	All	Aim 2	Aim 3	Aim 4
Participants, *n*	31*	**23**	**15**	**11**
Age, *years*	24.8 (22.7, 27.8)	24.9 (21.9, 27.9)	24.9 (23.3, 27.1)	26.1 (24, 28.1)
Height, *cm*	184 (176, 189.5)	183 (177, 190.8)	186 (177.5, 192)	188 (177.5, 193.5)
Weight, *kg*	81 (75, 85)	82 (76, 85.5)	82 (80, 92)	82 (81, 92)
BMI	24 (23, 26)	25 (23, 26)	25 (24, 26)	25 (14, 26)
Ethnicity				
Caucasian	25 (86%)	20 (87%)	14 (93%)	10 (91%)
Not provided	4 (14%)	3 (13%)	1 (7%)	1 (9%)
Matches played during study period				
Prior to first blood sample		4 (3, 9)	3 (3, 4)	3 (3, 5)
In month prior to first blood sample		3 (2, 3)	3 (2, 3)	3 (2, 3)
Between in-season and post-season blood sample		6 (4, 11)	10 (6, 11)	11 (8, 11)
Blood collection				
Time to sample post-match (h)		22 (19, 23)^+^	21 (18, 24)	20 (17, 23)
Years of collision sport participation				
0–5	0	0	0	0
6–10	3 (10%)	3 (13%)	0	0
11 +	26 (90%)	20 (87%)	15 (100%)	11 (100%)
History of concussion				
Yes	17 (59%)	12 (52%)	8 (53%)	6 (55%)
No	12 (41%)	11 (48%)	7 (47%)	5 (45%)
Number of previous concussions				
0	12 (41%)	11 (48%)	7 (47%)	5 (45%)
1	9 (31%)	8 (35%)	4 (27%)	3 (27%)
2	4 (14%)	2 (9%)	2 (13%)	2 (18%)
3 +	4 (14%)	2 (9%)	2 (13%)	1 (9%)

Data are presented as median (IQR) or *n* (%) where appropriate. *Two aim 1 participants did not complete the demographic questionnaire. ^+^Calculated for in-season collections only. *BMI* body mass index. All participants were male

### Head Impact Kinematics

**Fig 2.**
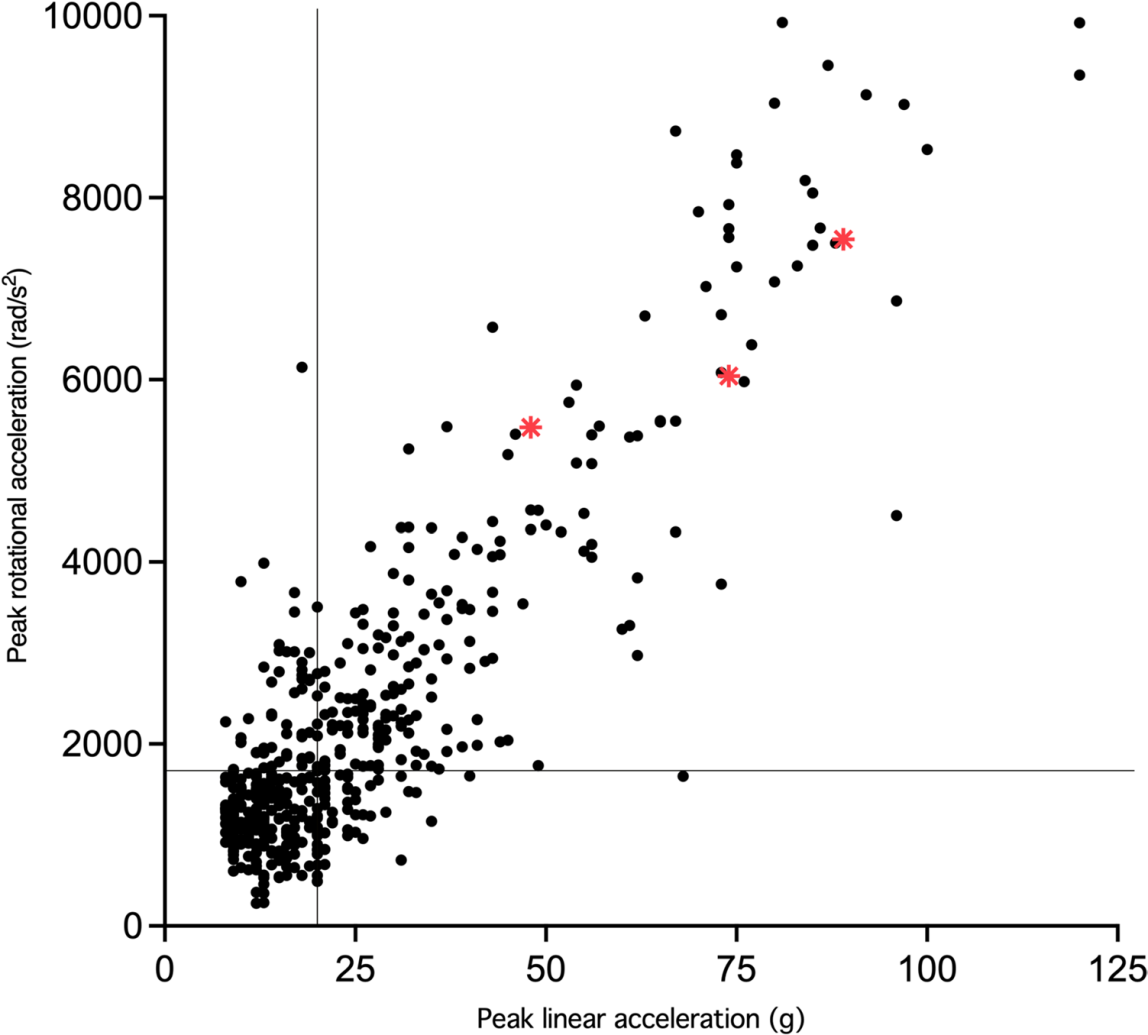
Video-verified true-positive head acceleration events in the study period (*n* = 484). Three participants sustained diagnosed concussions, with their linear and rotational acceleration data pairs denoted by red asterisks (*). These concussive impacts and corresponding blood collections were not included in any analyses. The median linear (20*g*) and rotational acceleration (1707 rad/s^2^) are denoted by the solid black lines

Across the 156 player matches, a total of 484 iMG captures above 8 g were video-verified true positive impacts (mean 3.10 impacts per game, median 2.44, IQR 2.04–3.36). For the 27 player matches used for paired HAE and biomarker data, the HAE frequency per match was comparable to the larger kinematic-only cohort (mean 3.41, median 3.00, IQR 1.00–3.00). The mean overall PPV was 86.4% (individual player median 91%, IQR 78–94%). A strong correlation was found between impact PLA and PRA for the 484 impacts (Fig. 2), with a median PLA of 20 g (IQR 13–32 g) and a median PRA of 1707 rad/s^2^ (IQR 1190–2899 rad/s^2^); three impacts to three individuals resulted in an SRC diagnosis (Fig. 2), with these PLA/PRA values and corresponding blood measures not included in blood analyses.

For our exploratory characterisation of head impact mechanisms in Australian football, analysis of play type revealed that tackles accounted for the highest number of HAEs (*n* = 202, 41%), with a similar number of HAEs resulting from the instrumented player tackling another person (*n* = 104, 51%) compared with the instrumented player being tackled (*n* = 98, 49%). Aerial (i.e. marking and ruck contests [38]) (*n* = 157, 32%) and player-to-player contact (e.g. bumps, fighting) (*n* = 87, 18%) accounted for the second- and third-highest number of HAEs, respectively.

### Blood Biomarkers

For in-season/post-match blood collections, the overall median time post-match was 22.00 h (IQR 19.00–23.42 h, min 16.83 h, max 27.00 h, mean 21.43 h, standard deviation 3.03 h; further details per aim in Table 1). For in-season samples, the median number of matches played prior to this blood collection was 4 (IQR 3–9, min 1, max 14, mean 6, standard deviation 4). For post-season samples, the median time following the final match of the season was 109 days (IQR 61–141 days, min 36 days, max 183 days, mean 103 days, standard deviation 46 days).

**Fig. 3:**
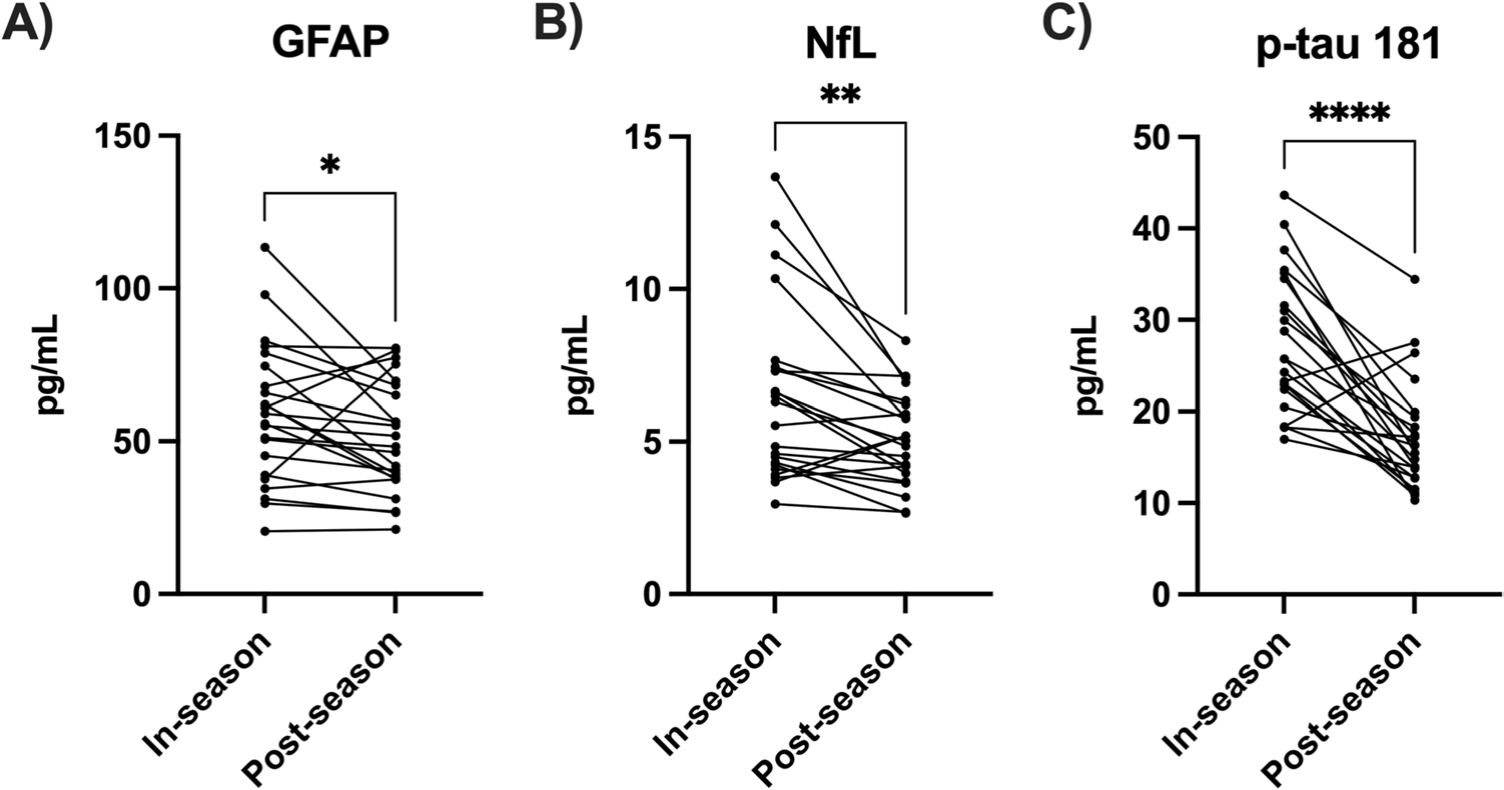
In-season post-match serum levels of GFAP (**A**) and NfL (**B**), and plasma levels of p-tau-181 (**C**), were significantly elevated compared to post-season levels in male amateur Australian footballers. Statistical analyses were performed with paired Student’s t-tests. *p<0.05, **p<0.01, ****p<0.0001

We firstly compared biomarkers in the 23 paired in-season and post-season samples (*n* = 22 for p-tau-181; one sample from an individual was incorrectly tested and consequently excluded). As shown in Fig. 3, in-season levels were higher than post-season for serum GFAP (Ln pg/mL mean difference 0.14, 95% CI 0.01–0.26, *p* = 0.033), serum NfL (Ln pg/mL mean difference 0.21, 95% CI 0.09–0.32, *p* = 0.001) and plasma p-tau-181 (Ln pg/mL mean difference 0.49, 95% CI 0.33–0.65,* p* < 0.001). Notably, paired in-season blood concentrations were higher than post-season for 18/23 individuals for GFAP, 19/23 for NfL and 20/22 for p-tau-181.

### Blood Biomarkers and Single Match Head Impact Kinematics

**Fig. 4:**
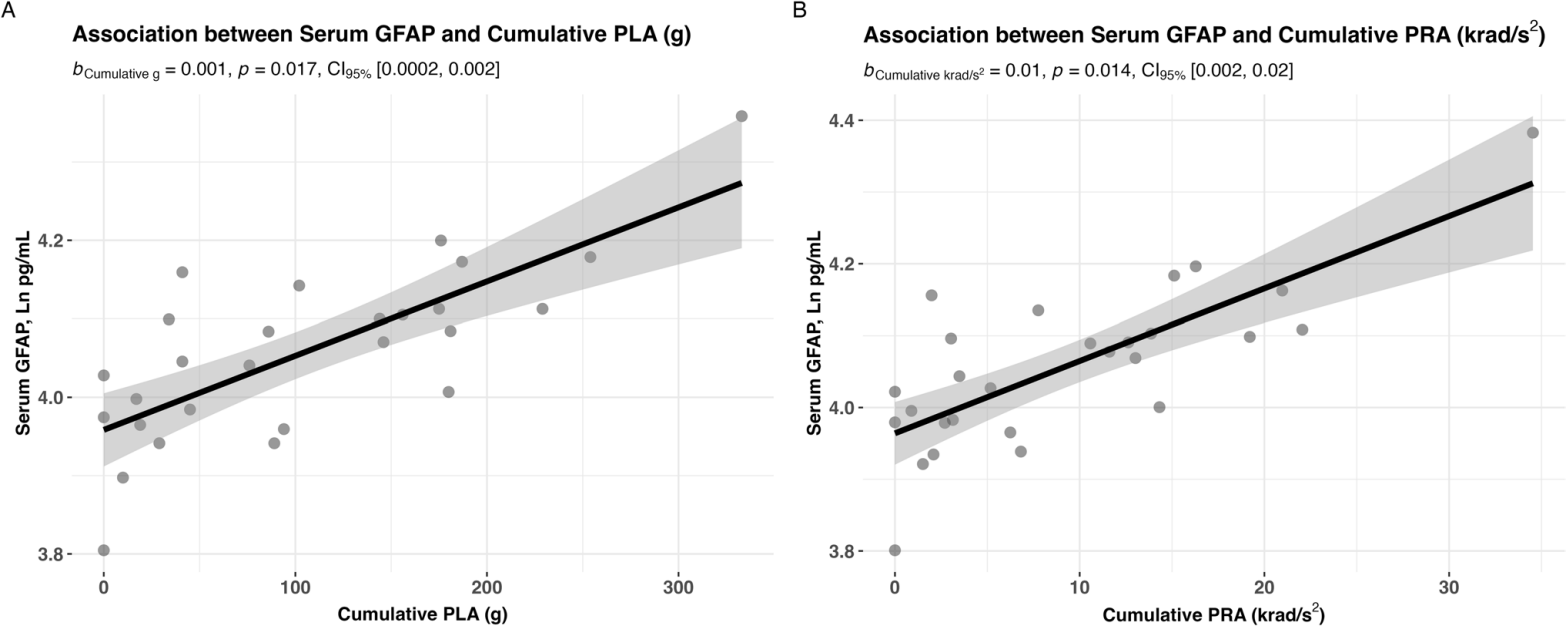
Post-match serum GFAP was significantly associated with the cumulative peak linear (**A**) and rotational (**B**) acceleration exposure in a single match of amateur Australian football. Statistical analyses were performed with linear mixed models with fixed effects of match number, age, and body mass index, and participant as a random term

Post-match biomarker (Ln transformed) and within-match cumulative PLA and PRA associations were assessed using linear mixed effect models. For 24 h GFAP levels (Fig. 4), we found a significant association between cumulative PLA (*B* = 0.001, 95% CI 0.0002–0.002, *p* = 0.017) and cumulative PRA (*B* = 0.01, 95% CI 0.002–0.02, *p* = 0.014). For NfL, no such association was seen for cumulative PLA (*B* = 0.0004, 95% CI − 0.0003 to 0.001, *p* = 0.265) and cumulative PRA (*B* = 0.003, 95% CI − 0.004 to 0.01, *p* = 0.372) (Online Resource 2). No association was found for p-tau-181 and cumulative PLA (*B* = 0.0004, 95% CI − 0.0005 to 0.001, *p* = 0.376) and cumulative PRA (*B* = 0.006, 95% CI − 0.003 to 0.02, *p* = 0.196) (Online Resource 3).

**Fig. 5:**
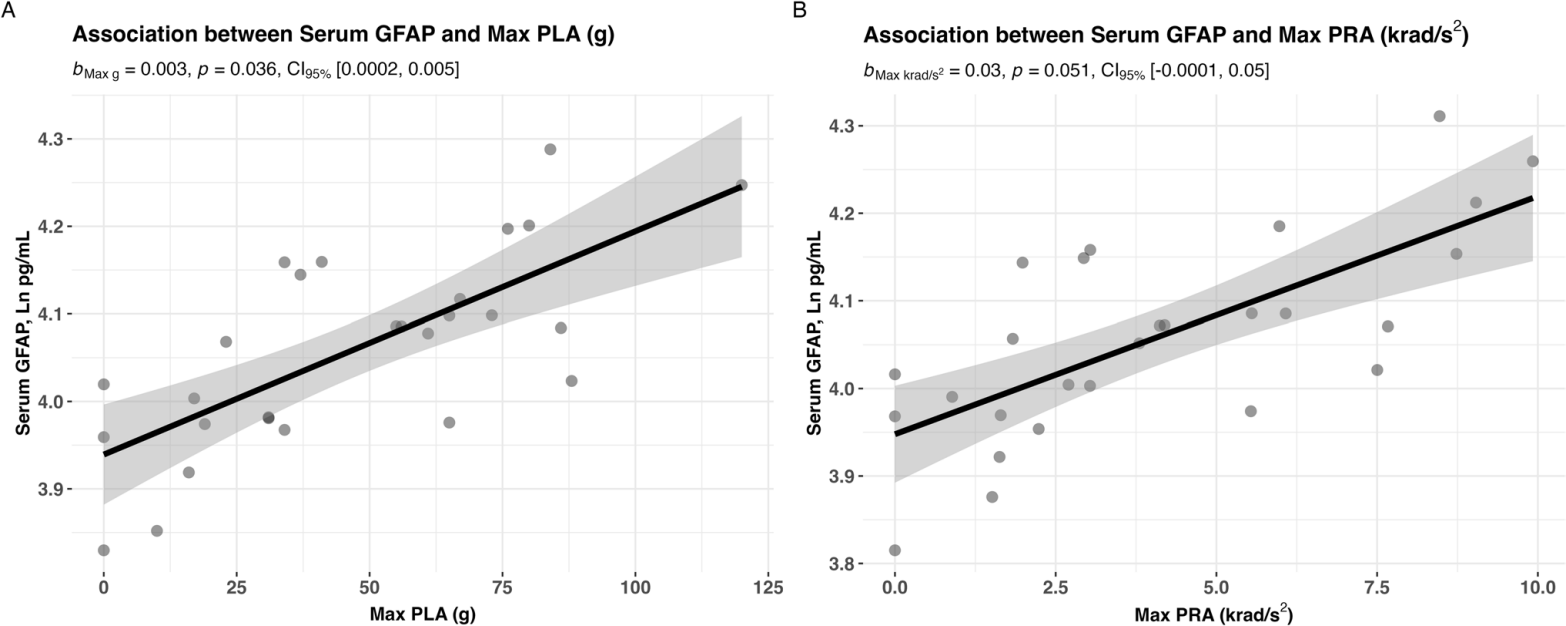
**A** Maximum peak linear acceleration was significantly associated with post-match serum levels of GFAP following a single match of amateur Australian football. **B** A non-significant trend was observed for an association between maximum peak rotational acceleration and post-match GFAP levels. Statistical analyses were performed with linear mixed models with fixed effects of match number, age, and body mass index, and participant as a random term

We next analysed the association between post-match biomarkers levels (Ln transformed) and the maximum PLA and PRA for a single impact (i.e. max PLA and max PRA). For GFAP at 24 h (Fig. 5), there was an association with max PLA (*B* = 0.003, 95% CI 0.0002–0.005, *p* = 0.036), and a trend for an association with max PRA (*B* = 0.03, 95% CI − 0.0001 to 0.05, *p* = 0.051). For NfL at 24 h, there was no association between max PLA (*B* = 0.0004, 95% CI − 0.002 to 0.003, *p* = 0.714) and max PRA (*B* = 0.007, 95% CI − 0.017 to 0.03, *p* = 0.551) (Online Resource 4). No associations were found for 24 h p-tau-181 and max PLA (*B* = 0.001, 95% CI − 0.002 to 0.004, *p* = 0.343) and max PRA (*B* = 0.02, 95% CI − 0.007 to 0.05,* p* = 0.131) (Online Resource 5).

Subsequently, we investigated the association between the number of head impacts within a single match and 24 h biomarker levels (Ln transformed). A significant association was found for GFAP (*B* = 0.03, 95% CI 0.003–0.05, *p* = 0.029), but not for NfL (*B* = 0.004, 95% CI − 0.02 to 0.03, *p* = 0.737) and p-tau-181 (*B* = 0.006, 95% CI − 0.02 to 0.04, *p* = 0.692) (Online Resource 6).

### Two-Match Change in NfL and Head Impact Kinematics

**Fig. 6:**
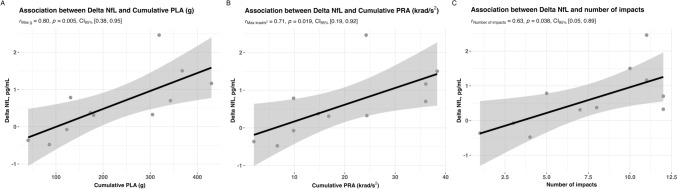
A significant association was observed between cumulative peak linear (**A**) and rotational (**B**) exposure, and the number of impacts (**C**), with changes in serum NfL levels within individuals across two consecutive games of amateur Australian football. Statistical analyses were performed with Spearman’s correlation analyses

Finally, we assessed the correlation between ΔNfL values (representing the difference between NfL post-match 2 and NfL post-match 1) and cumulative PLA, PRA, and impact number over the two-matches. For this ΔNfL value, a correlation was found for both cumulative PLA (Spearman’s *r* = 0.80, 95% CI 0.38–0.95, *p* = 0.005) and cumulative PRA (*r* = 0.71, 95% CI 0.19–0.92, *p* = 0.019) (Fig. 6A,B). In addition, ΔNfL values correlated with the total number of impacts over the two matches (*r* = 0.63, 95% CI 0.05–0.89, *p* = 0.038) (Fig. 6C).

## Discussion

This study provides novel evidence that links iMG-derived measures of single and repeated non-concussive head accelerations during a collision sport match to post-match increases in blood biomarkers indicative of brain cell injury. Specifically, we observed increased levels of serum GFAP and NfL, and plasma p-tau-181 during the in-season period compared with the post-season in male amateur Australian football players, with GFAP levels at 24 h post-match associated with within-match iMG measures of maximum single impact PLA, cumulative PLA and PRA, and number of impacts. While a similar relationship was not observed in post-match NfL and p-tau-181 levels and head impact kinematics in a single match, cumulative PLA, PRA, and number of impacts over two consecutive matches correlated with change in NfL concentration across this timeframe. These findings shed new light on how iMGs and blood biomarkers might be used to monitor head impact exposure within collision sports.

We identified consistent increases in blood levels of GFAP, NfL and p-tau-181 during the in-season period, in the absence of SRC, among male amateur Australian football players. While unique and noteworthy in this context, similar findings for GFAP and NfL have been reported in collegiate American football [19, 39]. The most striking of these in-season elevations in our cohort was for p-tau-181, possibly reflecting a transient increase in brain hyperphosphorylated tau due to HAEs. However, when considering evidence that recent physical activity may impact tau and p-tau-181 levels, potentially originating from peripheral sources [24, 29, 32], and considering our discovery of no correlation between HAE metrics and p-tau-181 levels, heightened in-season levels may primarily stem from the physical exertion endured during the match played 24 h earlier. Nonetheless, as the temporal profile of p-tau-181 after brain trauma and exercise are both poorly understood, future work is required to ascertain whether raised levels are associated with HAE exposure.

In contrast to p-tau-181, levels of the axonal cytoskeletal protein NfL in blood are unlikely to be affected by exercise alone [24, 28]. Therefore, the significant elevations in NfL observed in-season may be more reasonably attributed to collision sport HAE exposure. Furthermore, aligning with the kinetics of NfL after SRC [22, 27, 33], we found no association of 24 h NfL levels with HAE metrics. Instead, a notable correlation was found between the change in NfL levels and cumulative impact number, PLA, and PRA over a two-match period. These results indicate that repeated non-concussive head impacts sustained during Australian football match play can induce small degrees of axonal injury.

Regarding levels of astrocytic protein GFAP, we hypothesised that its kinetics, if similar to concussion, would favour measurement at 24 h post-HAE exposure, and that its shorter half-life would make it less likely for GFAP rises to summate across a season [33, 34]. Although we did find a subtle yet consistent elevation in GFAP levels in-season versus post-season, the apparent strong influence of recent collision sports exposure was supported by the associations between post-match GFAP and in-match acceleration metrics. Although the magnitude of GFAP elevation was, as expected, lower compared with mild TBI and SRC [14, 22, 26, 27], the finding that levels were elevated in-season and were associated with various head kinematic measures supports the hypothesis that non-concussive impacts can induce neurobiological changes.

Our findings offer insights into both the potential cumulative effects of repeated head impacts and the isolated effects of a single high-force head impact. We found associations between both the cumulative and maximum accelerations and GFAP levels, with the latter association providing evidence supporting the need for closer examination of high-force impacts, even in the absence of clear signs and symptoms. It is important to acknowledge that single high-magnitude impacts were included in cumulative acceleration totals, therefore it is possible biomarker elevations were driven by these maximum impacts. Nonetheless, there was an association between post-match GFAP and number of impacts in a single match, and the change in NfL levels over a two-match period was associated with both cumulative acceleration load and total number of impacts sustained in the two matches. Considered together, while our study indicates that single high-acceleration impacts may induce rises in blood GFAP, a larger sample size and more nuanced analytical approaches are required to tease apart their contribution to neurobiological changes relative to a series of smaller magnitude impacts. Similarly, while there is evidence that rotational acceleration may be more likely than linear acceleration to result in brain tissue strain, our study found biomarker associations with both forms of acceleration, and future studies are required to understand their relative contribution to biomarker elevations.

This study provides new insights into how head impact management could be enhanced by implementing iMGs and blood biomarker measures. Regarding the use of blood biomarkers, our findings support the use of both GFAP and NfL, but likely in different situations. For probing the potential neurobiological effects of HAE exposure within a single collision sport session, measures of GFAP at or around 24 h post-session may be most suitable. In contrast, NfL may be best suited for assessing HAEs over a more prolonged period, with its longer half-life creating a larger window for such insights. Nevertheless, these damage-associated markers are of different cellular origin (GFAP from astrocytes in the brain and spinal cord and NfL from axons in the brain, spinal cord, and peripheral nerves), therefore it is possible that one marker may be more sensitive and specific. Related to this, while some have shown an association between HAE exposure and tau and S100B, these markers have relatively lower brain specificity when compared with GFAP and NfL [32], and both have been shown in multiple studies to be elevated after exercise [24, 29, 30]. Therefore, the specificity of GFAP and NfL for brain trauma, especially in young adults where neurodegenerative conditions that could complicate interpretation are uncommon, emphasises their suitability for integration into the framework of head impact management in collision sport.

Our research yields strong evidence supporting the neurobiological correlation with iMG measures of non-concussive HAEs within collision sports. This endorsement supports the adoption of iMG technology for monitoring individual head impacts, irrespective of clinical signs and symptoms, and for tracking cumulative HAE burden in a training session, match, or season. The optimal head impact management strategy is likely to integrate both iMG and biomarkers. This approach may reduce the occurrence of false positives. For instance, elevated NfL levels in the absence of HAEs may indicate a peripheral nerve injury (e.g. following major musculoskeletal injury [40]), whereas an individual sustaining high-acceleration impacts or accumulating substantial loads may have a relatively high biomechanical tolerance and therefore exhibit no biomarker elevations. Related to this, despite our results revealing no clear biomechanical thresholds for biomarker elevations or SRC risk, a more extensive sample size, including a greater number of impacts and more than three SRC cases, is essential to assess selection of thresholds and their resultant sensitivity and specificity. The idea of employing iMG-derived acceleration thresholds in real-time during matches to trigger concussion screening is undeniably appealing. Our findings support the expansion of this suggested screening approach beyond evaluating the clinical features of SRC to encompass post-match blood measures of GFAP and NfL. This inclusion would enhance the overall comprehensiveness of head impact management protocols.

This study had several limitations. First, despite the targeted multimodal, accurate and blinded measures, the sample of this study was relatively small and derived from a convenience sample rather than an a priori sample size calculation. Consequentially, a potential lack of power can be seen in the wide confidence intervals. Future studies in larger cohorts that involve female athletes and children are required to validate findings and their generalisability. We did not perform multiple comparison corrections due to the distinct hypotheses for GFAP, NfL, and p-tau-181, each reflecting different aspects of pathophysiology. This approach may increase the risk of Type I errors. While SRC signs and symptoms were not reported for all impacts in this study, nor observed during blinded video review, the subjective nature of concussion signs and symptoms means that some cases of SRC may have been missed. Matches were filmed using only one camera angle, with some variation in footage quality. As a result, some impacts were unable to be video verified. While each impact was verified twice, all impacts were coded by a single rater. Related to this, our data show that tackles appear to be a primary cause of HAEs in Australian football; however, more detailed coding of larger and broader cohorts is required to increase understanding of HAEs at different levels of this widely participated-in collision sport. Additionally, while a 24 h post-match sampling period may be optimal for GFAP, later timepoints might be more appropriate for detecting NfL rises. The timing for detecting elevated p-tau-181 levels following brain trauma remains unclear, though more acute measures might be optimal. Furthermore, although our sampling window was narrow, variations in time to sample could have influenced the findings. The absence of a non-collision sport control group is a limitation, primarily for aim 2, as we cannot account for factors beyond HAEs that might have contributed to the observed elevated levels of biomarkers in the in-season compared with the post-season period. Although the associations between iMG-derived metrics and GFAP and NfL suggest that head impacts are likely a significant contributor in these elevations, other factors, such as variations in exercise intensity, may also play a role. While GFAP and NfL are highly brain-specific and their subtle elevations in blood likely reflect a small degree of brain cell damage, the potential consequences of these elevations in asymptomatic individuals remain unclear. Future studies are needed to determine whether subtle elevations are clinically actionable, such as prompting removal from play or recommending rest. Although it is reasonable to speculate that prolonged exposure to HAEs at levels sufficient to cause biomarker changes may have consequences for brain structure and function, longitudinal studies involving neuroimaging and functional outcomes are necessary to explore these potential effects and assess whether management strategies guided by iMG or biomarker use can prevent them.

## Conclusions

In this study with male amateur Australian football players, we found a significant association between iMG-measures of maximum and cumulative non-concussive head impact accelerations during a match and elevated blood biomarkers indicative of brain cell injury. While future research is needed to establish HAE thresholds and to define clinically significant biomarker changes, these findings highlight the potential benefits of integrating both iMGs and blood biomarkers for improved head impact management in collision sports.

## Supplementary Information

Below is the link to the electronic supplementary material.

Supplementary file1 (DOCX 2966 kb)

## Data Availability

Data are available upon reasonable request to the corresponding author.
