# Supplementary material for: Associations Between Instrumented Mouthguard-Measured Head Acceleration Events and Post-Match Biomarkers of Astroglial and Axonal Injury in Male Amateur Australian Football Players
Source: Sports Med. 2024 Nov 19;55(4):1037–49. doi: 10.1007/s40279-024-02138-6 (PMC12011967; doi:10.1007/s40279-024-02138-6)

**Article title:** Associations Between Instrumented Mouthguard-Measured Head Acceleration Events and Post-Match Biomarkers of Astroglial and Axonal Injury in Male Amateur Australian Football Players

**Journal name:** Sports Medicine

**Authors:** Lauren J. Evans*^1^, William T. O’Brien*^1^, Gershon Spitz^1,2^, Steven Mutimer^1^, Becca Xie^1^, Lauren P. Giesler^1^, Brendan P. Major^1^, James W. Hickey^1^, Spencer S. H. Roberts^3^, Biswadev Mitra^4,5^, Terence J. O’Brien^1,6,7^, Sandy R. Shultz^1,6, 8^, Stuart J. McDonald^^1, 6^

**Affiliations:**

^1 Department of Neuroscience, Central Clinical School, Monash University, 99 Commercial Road, Melbourne, VIC, Australia^

^2 Monash-Epworth Rehabilitation Research Centre, School of Psychological Sciences, Monash University, Clayton, VIC, Australia^

^3 School of Exercise and Nutrition Sciences, Deakin University, Burwood, VIC, Australia^

^4 Emergency & Trauma Centre, The Alfred Hospital, Melbourne, VIC, Australia^

^5 School of Public Health and Preventive Medicine, Monash University, Melbourne, VIC, Australia^

^6 Department of Neurology, The Alfred Hospital, Melbourne, VIC, Australia^

^7 Department of Medicine, Royal Melbourne Hospital, The University of Melbourne, Parkville, VIC, Australia^

^8 Centre for Trauma & Mental Health Research, Vancouver Island University, Nanaimo, BC, Canada^

* Co-first authors ^ Corresponding author

**Corresponding Author:** Stuart J. McDonald, Department of Neuroscience, Central Clinical School, Monash University, 99 Commercial Road, Melbourne, VIC, 3004, Australia. Phone: +61 3 9903 0203; Fax: +61 3 9903 0843; E-mail: stuart.mcdonald@monash.edu

| **2022** | **Apr**  **2** | **Apr**  **9** | **Apr 23** | **Apr**  **30** | **May 7** | **May 14** | **May**  **21** | **May**  **28** | **Jun**  **4** | **Jun**  **11** | **Jun**  **18** | **Jun 25** | **Jul**  **2** | **Jul**  **9** | **Jul**  **16** | **Jul**  **23** | **Jul**  **30** | **Aug**  **6** | **Aug**  **13** | **Aug**  **20** | **Aug**  **27** | **Sept 3** | **Sept 10** | **Sept 17** |
| --- | --- | --- | --- | --- | --- | --- | --- | --- | --- | --- | --- | --- | --- | --- | --- | --- | --- | --- | --- | --- | --- | --- | --- | --- |
| P1 |  |  | MG | MG | MG | MG | MG |  | MG |  | MG | MG |  |  | MG | MG |  | MG | **W** |  |  |  |  |  |
| P2 | P | P | MG | **MG** | MG | MG |  |  |  |  | MG |  |  |  |  |  |  |  |  |  |  |  |  |  |
| P3 | P | P | MG | **MG** | MG | MG | MG |  | MG |  | MG | MG |  |  | MG | MG |  | MG | MG | MG |  | MG | MG | MG |
| P4 | P | P | MG | **MG** | MG | P | MG |  | MG |  |  |  |  |  |  |  |  | MG |  |  |  |  |  |  |
| P5 | P | P | MG | **MG** | MG |  | MG |  |  |  |  |  |  |  |  |  |  |  | MG | MG | MG |  |  |  |
| P6 | P | P | MG | MG | **MG** | MG | MG | MG | MG |  | MG | MG |  |  | MG | MG |  | MG | MG |  | MG |  |  |  |
| P7 | P | P | MG | **P** | MG | P | MG | MG | MG |  | MG | MG |  |  | MG |  |  |  |  |  | MG |  |  |  |
| P8 | P | P | MG | MG | **W** |  |  |  |  |  |  |  |  |  |  |  |  |  |  |  |  |  |  |  |
| P9 | P | P | MG | **MG** |  |  | MG |  | MG |  | MG | P |  |  |  |  |  |  |  |  |  |  |  |  |
| P10 | P | P | MG | MG | MG | P | MG | MG | MG |  | MG | MG |  |  | MG | MG | MG | MG | MG |  | MG |  |  |  |
| P11 | P | P | MG | MG | MG | MG | MG | MG | MG |  | MG |  |  |  | MG | MG | MG | MG | MG | P | MG |  |  |  |
| P12 | P | P |  | MG | P | MG | MG | **MG** | MG |  | MG | MG |  |  | MG | MG | MG | P | MG | P | MG |  |  |  |
| P13 | P | P |  |  | **MG** | MG | MG | MG |  |  |  |  |  |  | MG | MG | MG | MG | MG | P | P |  |  |  |
| P14 |  |  |  |  | MG | **MG** | MG |  | MG |  | MG | MG |  |  | MG | MG |  | MG | MG |  | MG |  |  |  |
| P15 |  | P | MG | MG | **MG** | MG |  | MG | MG |  | MG | MG |  |  | MG | MG |  |  | MG |  |  | MG | MG | MG |
| P16 | P | P | P | MG | **MG** | MG | MG |  | MG |  | MG | MG |  |  | MG | MG |  | MG | MG | MG | MG |  |  |  |
| P17 | P | P | MG |  | MG | MG | MG | **W** |  |  |  |  |  |  |  |  |  |  |  |  |  |  |  |  |
| P18 | P | P |  |  | P | P | P | P | P |  | P | **P** | P |  | P |  |  |  | P | P |  |  |  |  |
| P19 |  | P | P | P | P | P | P |  | P |  | P | **P** |  |  | P | P | P | P |  | P |  |  |  |  |
| P20 | P | P | P | P |  | P | P | P | P |  | P | **P** |  |  | P | P | P | P | P | P |  |  |  |  |
| P21 |  |  |  |  |  | MG | MG |  | MG |  | MG | MG |  |  | MG | **MG** |  | MG | MG | MG | MG | MG | MG | MG |
| P22 | P | P | P | P | P | P | P | P | P |  | P | P | P |  | P | **P** | P | P | P | P |  |  |  |  |
| P23 | P | P | P |  | P | P |  | P | P |  | P |  | P |  |  | P | **P** |  | P |  |  |  |  |  |
| P24 |  |  |  |  |  |  |  | P | P |  | P |  |  |  |  | P | **P** | P | P | P | P |  |  |  |
| P25 | P | P |  | P |  | P | MG | MG | MG |  | MG |  |  |  |  | MG | MG | **MG** | MG | P | MG |  |  |  |
| P26 |  |  |  |  |  |  | MG |  | MG |  | MG |  |  |  |  |  |  | MG | **MG** | MG | MG | MG | MG | MG |
| P27 | P | P | MG |  |  |  | MG | MG | MG |  |  |  |  |  | MG | MG | MG | MG | MG | P | MG |  |  |  |
| P28 |  |  |  |  |  |  |  |  |  |  |  |  |  |  |  |  |  |  | P | **P** |  |  |  |  |
| P29 | P | P | P | P | P | P | P | P |  |  |  |  |  |  | P | P | P | P | P | **P** |  |  |  |  |
| P30 |  |  |  | MG | MG | MG | MG |  |  |  |  |  |  |  | MG | MG |  |  |  |  |  |  |  |  |
| P31 |  |  | MG |  | MG |  | MG | MG | MG |  | MG | MG |  |  | MG | MG | MG | MG | MG | P | MG |  |  |  |

**Online Resource 1. Participant post-match collections across 2022 VAFA season.**

**P1-31:** Participant 1-31.

**P**: Played a match, but either did not wear iMG or data was not recorded. (No player wore the MG before Apr 23).

**MG**: Played a match wearing iMG and data recorded.

**W**: Player withdrawn from this point onwards.

**Pink shading**: Post-match blood collection (pre-organised as part of BIOREC study - O’Brien et al 2024; <https://jamanetwork.com/journals/jamanetworkopen/fullarticle/2819700>)

**Dark red shading**: Aim 2 in-season post-match blood collection.

Date represents the Saturday of each week.

**Online Resource 2.** Cumulative peak linear and peak rotational acceleration exposure in a single game was not associated with post-match serum NfL levels.


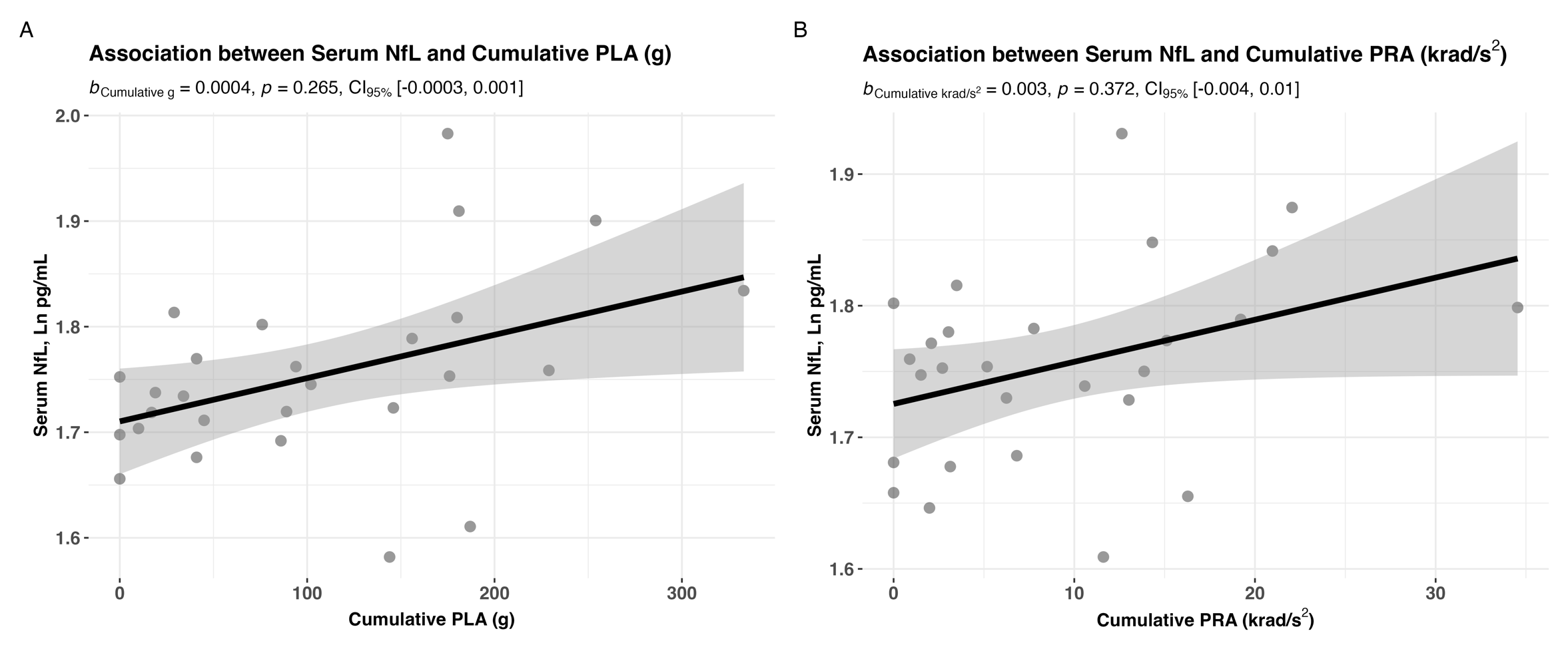


**Online Resource 3.** Cumulative peak linear and peak rotational acceleration exposure in a single game was not associated with post-match plasma p-tau-181 levels.


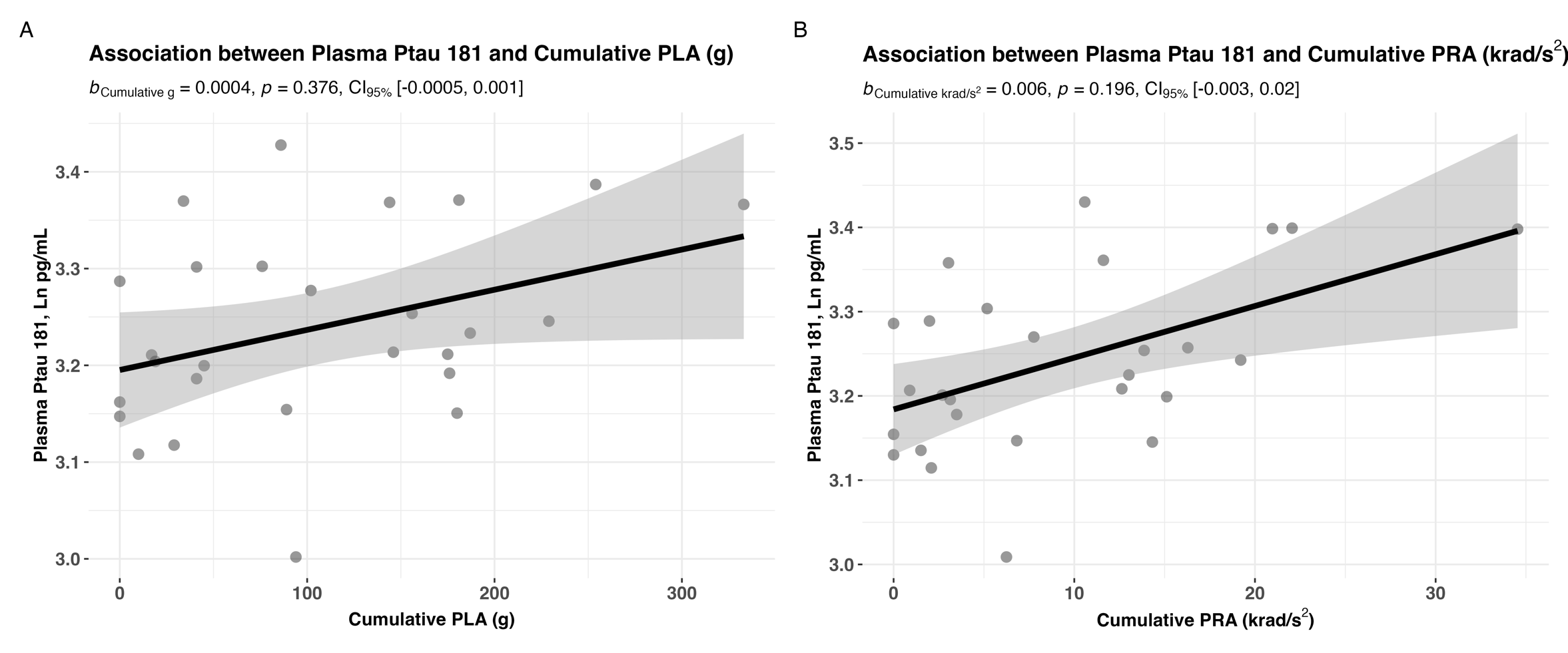


**Online Resource 4.** Maximum peak linear and peak rotational acceleration exposure in a single game was not associated with post-match serum NfL levels.


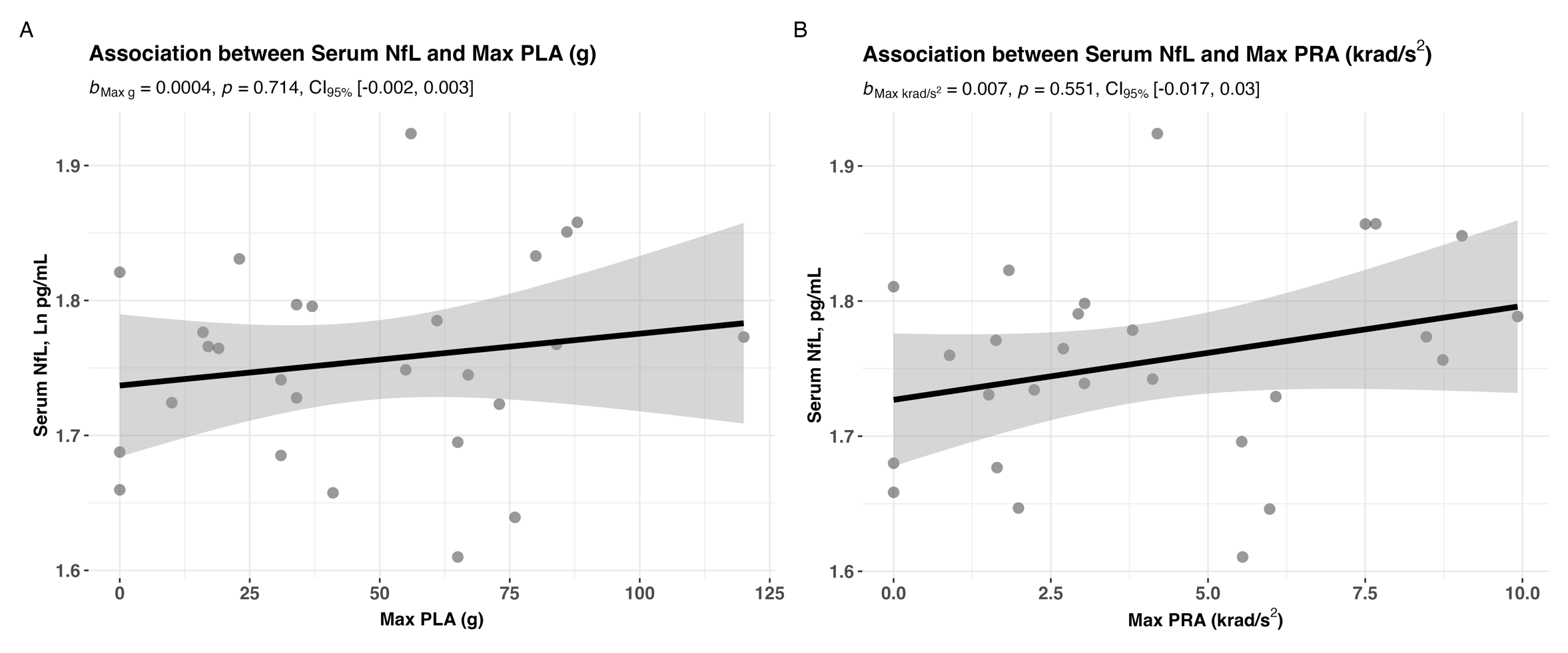


**Online Resource 5.** Maximum peak linear and peak rotational acceleration exposure in a single game was not associated with post-match plasma p-tau-181 levels.


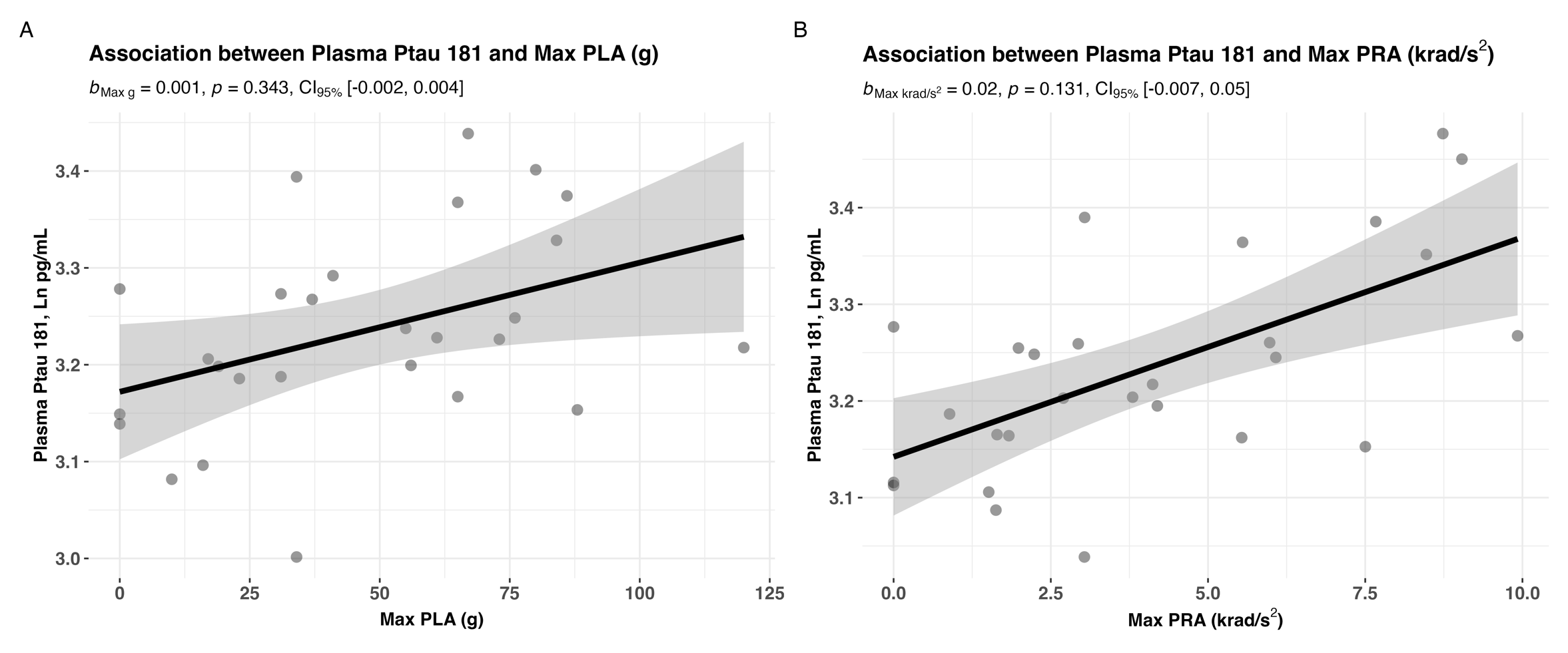


**Online Resource 6.** A significant association was observed between post-match serum GFAP (A), but not serum NfL (B) nor plasma p-tau-181 (C), and the number of impacts sustained during a single game of amateur Australian football.


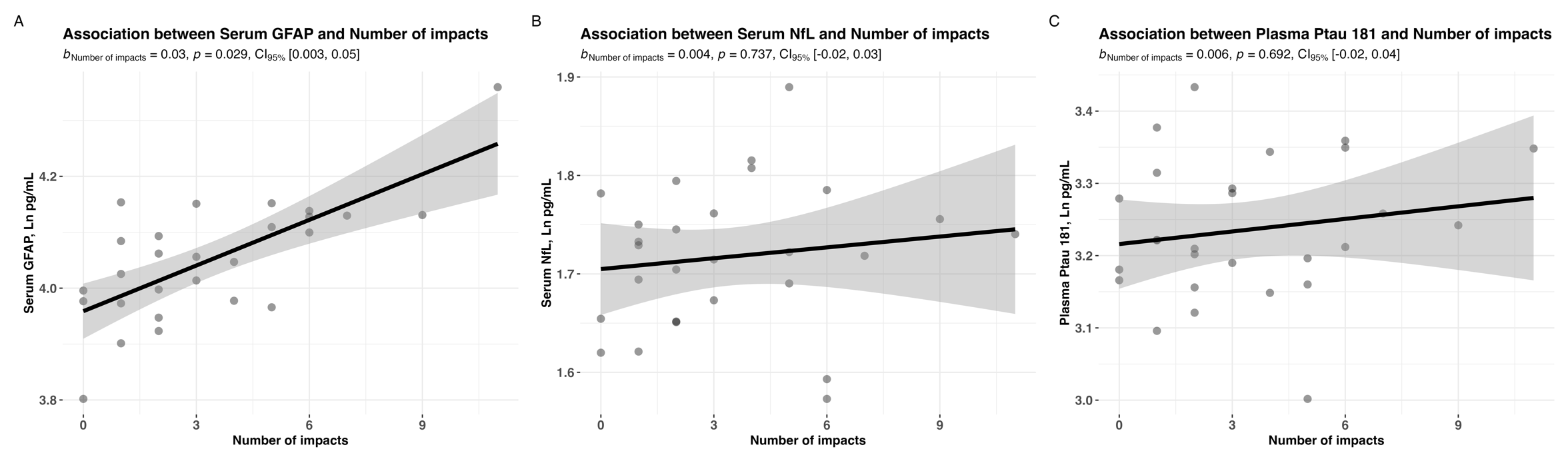

Supplement: Supplementary file 1 — Supplementary file1 (DOCX 2966 kb) [file 40279_2024_2138_MOESM1_ESM.docx]
